# Content Validation of an Electronic Health Record–Based Diabetes Self-Management Support Tool for Older Adults With Type 2 Diabetes: Qualitative Study

**DOI:** 10.2196/83448

**Published:** 2026-02-06

**Authors:** Ploypun Narindrarangkura, Siroj Dejhansathit, Uzma Khan, Margaret Day, Suzanne A Boren, Eduardo J Simoes, Min Soon Kim

**Affiliations:** 1 Phramongkutklao College of Medicine, Bangkok, Thailand; 2 Cosmopolitan International Diabetes and Endocrinology Center, University of Missouri, Columbia, MO, United States; 3 Department of Medicine, University of Missouri, Columbia, MO, United States; 4 Department of Family and Community Medicine, University of Missouri, Columbia, MO, United States; 5 University of Missouri Institute for Data Science and Informatics, Columbia, MO, United States; 6 Department of Health Sciences, College of Health Sciences, University of Missouri, 310 Clark Hall, Columbia, MO, 65211, United States, 1 5738840115; 7 Department of Biomedical Informatics, Biostatistics and Medical Epidemiology, University of Missouri, Columbia, MO, United States

**Keywords:** diabetes mellitus, type 2, electronic health records, self-management, patient education, older adults, digital health, health literacy

## Abstract

**Background:**

Older adults with diabetes frequently access their electronic health record (EHR) notes but often report difficulty understanding medical jargon and nonspecific self-care instructions. To address this communication gap, we developed Support-Engage-Empower-Diabetes (SEE-Diabetes), a patient-centered, EHR-integrated diabetes self-management support tool designed to embed tailored educational statements within the assessment and plan section of clinical notes.

**Objective:**

This study aimed to validate the clarity, relevance, and alignment of SEE-Diabetes content with the Association of Diabetes Care & Education Specialists 7 Self-Care Behaviors framework from the perspectives of older adults and clinicians.

**Methods:**

An interdisciplinary team conducted expert reviews and qualitative interviews with 11 older adults with diabetes and 8 clinicians practicing in primary care (family medicine) and specialty diabetes care settings at a Midwestern academic health center. Patients evaluated the readability and relevance of the content, while clinicians assessed clarity, sufficiency, and potential clinical utility. Interview data were analyzed using inductive thematic analysis, and descriptive statistics were used to summarize participant characteristics.

**Results:**

Patients (mean age 72, SD 4.9 y; mean diabetes duration 26, SD 15 y) reported that the SEE-Diabetes statements were clear, relevant, and written in plain language that supported understanding of self-care recommendations. Clinicians (mean 13, SD 9.5 y of diabetes care experience) viewed the content as concise, clinically appropriate, and well aligned with patient self-management goals and the Association of Diabetes Care & Education Specialists 7 Self-Care Behaviors framework. Both groups identified the tool’s potential to enhance patient engagement and patient-clinician communication, while noting opportunities to improve the specificity of language, particularly within medication-related content.

**Conclusions:**

SEE-Diabetes demonstrated content validity as a practical, patient-centered digital health tool for supporting diabetes self-management communication within EHR clinical notes. The findings support its use as a complementary approach to reinforce self-care communication in routine clinical practice and highlight areas for refinement to enhance personalization.

## Introduction

### Background

Diabetes is highly prevalent among older adults in the United States, with an estimated 29.2% of adults aged 65 years or older having been diagnosed with or undiagnosed diabetes during 2017‐2020, and approximately 48.8% of adults in this age group had prediabetes according to the most recent National Diabetes Statistics Report [1]. As the aging population grows, primary care clinicians face increasing pressure to deliver effective, individualized diabetes self-management education within routine visits. Diabetes self-management education and support (DSMES) has been shown to improve glycemic control, reduce complications, and enhance self-efficacy [2-5]. However, the delivery of DSMES in outpatient settings is frequently constrained by limited visit time, complex documentation requirements, challenges in referral and access, and poor integration with routine clinical workflows [6,7].

National DSMES standards outline 4 critical times when individuals with diabetes should receive structured education and support [2]; however, referrals and access to formal DSMES services remain inconsistent. As a result, self-management guidance is often delivered informally during routine visits, underscoring the need for tools that reinforce evidence-based messaging within existing clinical workflows.

To address these challenges, our team developed Support-Engage-Empower-Diabetes (SEE-Diabetes), a patient-centered educational aid designed to support clinicians in delivering tailored diabetes education to older adults during clinic visits. SEE-Diabetes integrates directly into the electronic health record (EHR) by embedding brief, personalized education statements—drawn from a curated content library—into the assessment and plan section of the clinician’s note. The content is organized according to the 7 core domains of the Association of Diabetes Care & Education Specialists 7 Self-Care Behaviors (ADCES7), including healthy coping, healthy eating, being active, taking medication, monitoring, reducing risk, and problem solving [8].

Placement of SEE-Diabetes in the Assessment and Plan section was intentional. Prior formative research with older adults with diabetes from our group found that the majority (80%) accessed and read their clinic notes through patient portals, yet many found these notes difficult to understand due to medical jargon and vague or nonactionable self-care guidance [6,7]. Embedding clear, relevant, and actionable statements in a section that patients already read may therefore address an important communication gap while also integrating seamlessly into clinician documentation.

SEE-Diabetes was developed using a user-centered design (UCD) approach to ensure alignment with real-world clinical needs [9,10]. The first stage of development involved an analysis of EHR documentation patterns related to diabetes care [11], followed by a second stage comprising focus groups with older adults with type 2 diabetes and clinicians involved in diabetes management to identify gaps in the clarity, readability, and consistency of self-management information [6,7]. This study represents the third stage of the UCD process and focuses on content validation of the SEE-Diabetes educational statements to ensure their accuracy, relevance, and practical utility for both patients and clinicians [12].

### Objective

Our objective was to assess the clarity, helpfulness, and perceived value of SEE-Diabetes education content by conducting in-depth interviews with older adults and clinicians practicing in primary care (family medicine) and specialty diabetes care settings. This validation step is essential before the broader implementation of SEE-Diabetes in primary care settings. By embedding actionable, comprehensible diabetes education into clinical notes, SEE-Diabetes may enhance patient understanding, improve continuity of care, and support more effective chronic disease management among older adults.

## Methods

### Study Design

We conducted a qualitative content validation study to assess the clarity, readability, and clinical relevance of SEE-Diabetes, an EHR-integrated education tool for older adults with diabetes. This phase represented the third stage of a UCD process. The content validation process included (1) expert reviews by clinicians and certified diabetes care and education specialists, and (2) user feedback through semistructured interviews with older adults with diabetes and with primary care or endocrinology clinicians. The interdisciplinary research team included experts in informatics, endocrinology, primary care, and diabetes education.

### Description of SEE-Diabetes

**Figure 1.**
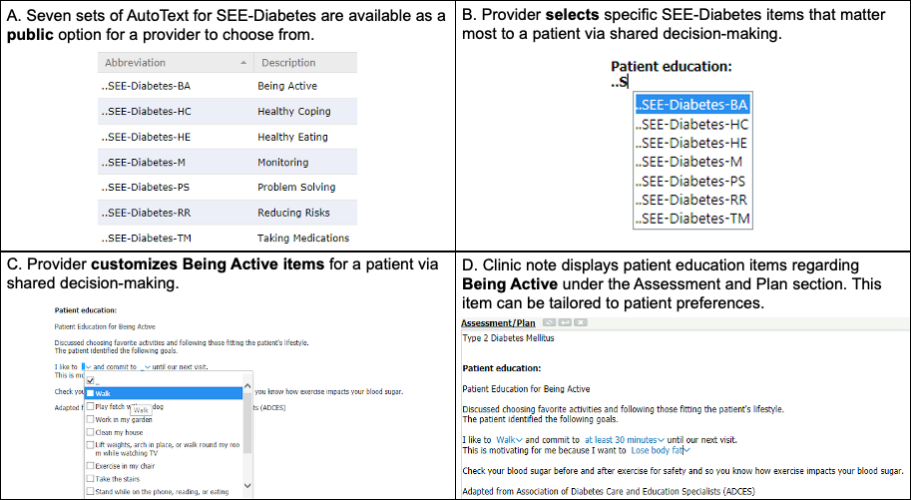
Overview of the Support-Engage-Empower-Diabetes framework illustrating integration of tailored patient education statements into electronic health records, aligned with the Association of Diabetes Care & Education Specialists 7 Self-Care Behaviors. (A) Seven publicly available autotext sets (being active, healthy coping, healthy eating, monitoring, problem solving, reducing risks, and taking medications) are mapped to Association of Diabetes Care & Education Specialists 7 Self-Care Behavior domains. (B) The clinician selects the relevant Support-Engage-Empower-Diabetes category in the Patient education field. (C) Within the chosen category, statements are customized collaboratively (eg, activity type, frequency, or targets) during shared decision-making. (D) The finalized, tailored patient education statements are inserted into the Assessment and Plan section of the clinic note and become available to patients via the portal. ADCES7: Association of Diabetes Care & Education Specialists 7 Self-Care Behavior; SEE-Diabetes: Support-Engage-Empower-Diabetes.

SEE-Diabetes content was implemented within the EHR as “auto-text” templates in Oracle Cerner’s PowerChart. During documentation, the clinician first selects the SEE-Diabetes category most relevant to the patient’s needs, informed by shared decision-making during the visit. Within the category chosen, the clinician can review and select multiple educational statements addressing specific self-care behaviors. Each statement can be further customized to reflect the patient’s individual preferences, goals, literacy level, and clinical circumstances. Examples of customization include changing the activity type (eg, walking and gardening) or specifying behavior targets (eg, number of minutes per day) (Figure 1).

The finalized patient education text was embedded within the assessment and plan section of the clinic note. Embedding SEE-Diabetes in the assessment and plan positions the guidance where patients already expect to find follow-up instructions, while requiring minimal change to clinician workflow. Insertion and customization generally take less than 1 minute, minimizing any disruption to the visit flow.

### Study Setting

The study was conducted at the University of Missouri Health Care, an academic medical center serving 114 counties in Missouri [13]. The center uses the Oracle Cerner PowerChart EHR system to consolidate patient data across facilities. Patients can access their medical records, including clinic notes, through the HEALTHConnect portal. Clinic notes were retrieved from PowerChart, and patient recruitment was facilitated using PowerInsight, Oracle Cerner’s operational reporting platform.

### Interview Development

A total of 3 representative clinical scenarios were developed based on de-identified data from older adults with type 2 diabetes. For each case, SEE-Diabetes was applied to generate tailored patient education statements aligned with ADCES7 domains. The scenarios were (1) a 69-year-old woman with uncontrolled diabetes (monitoring and healthy eating), (2) a 72-year-old man with stable diabetes and obesity (medication adherence and risk reduction), and (3) a 67-year-old woman with type 2 diabetes (physical activity and healthy coping). An endocrinologist drafted the clinic notes (history of present illness and assessment and plan), and the multidisciplinary team reviewed all content for clinical accuracy and guideline concordance. The history of present illness sections are shown in Textbox 1.

Textbox 1. **History of present illness section of three clinic notes. These are in-model screenshots of three example clinic notes designed for patients with diabetes aged 65 years and older attending follow-up visits at the Cosmopolitan International Diabetes and Endocrinology Center. Participants were asked to review the history of present illness section along with the assessment and plan.**

**Clinic Note 1: History of Present Illness**

- 69-year-old lady presents for discussion regarding long-term management of diabetes mellitus.
- Initially diagnosed in 2009,

- started on metformin 1000 mg BID
- glimepiride 4 mg BID started in 2010
- pioglitazone 45 mg QD in the morning started in 2019
- has never been on insulin

- She has not had any diabetic education since her diagnosis. Denies numbness/tingling in her extremities. She has tried a keto diet in the past, but this led to frequent hypoglycemia. Since July 2022, she has been eating <1700 calories daily, which resulted in weight gain. Eye exam was done in April 2022 but not sure if her eyes were dilated. She has noted that her vision changes as her BG fluctuates, and sometimes her vision is blurry despite wearing her bifocals. There is no family history of T2DM, T1DM, or osteoporosis, and has never had a DEXA scan. She sees gynecologist yearly but does not have a regular well-woman exam.

**Clinic Note 2: **History of Present Illness

- This is a 72-year-old gentleman who presents for follow-up of his diabetes.
- He was diagnosed with diabetes around the age of 50 and has been on metformin since that time.
- Blood glucose is slightly worse; he checks every day and has been running above 150 mg/dl
- He is on Metformin 1000 mg twice a day. He has noted increased blood glucose since he got Covid in 1/2022.
- He has had fatigue, feels nauseous, so has not been taking his metformin daily, only taking it "on good days."
- He was supposed to meet with a dietitian, but has had so many doctors' appointments that did not make it.
- His family doctor added a "small pill every day," but he is not sure what medication it is.
- He does not want to use any medications that are injections at this time and feels he can control his diabetes once he is feeling better.
- His HbA1c_1c_ was 7.5%; it has increased now to 8.8%.
- He plans on focusing on lifestyle and has been having increased burning in feet, so he was not walking much.

**Clinic Note 3: History of present illness**

- 67-year-old patient who presents to discuss diabetes mellitus type 2 management
- DMT2 diagnosed age 55 years. There is no retinopathy, neuropathy; she has microalbuminuria. She also has hyperlipidemia and hypertension
- Current regimen includes glipizide 10 mg daily and metformin extended release 500 mg, takes 1 tablet twice a day
- Her last diabetes class was before 2014
- Checks FSG once a day, ranges from 122-140s, no hypoglycemia
- She was walking, had to quit because of arthritis, now spends most of her time at home, and feels discouraged about her diabetes
- She likes to bake but has no motivation to do it anymore. Three friends have passed away in the last four years, and she has no family near home. She tries to eat healthy, mainly frozen meals.
- She is a smoker, has been trying to quit but feels she cannot do it.
- Blood pressure had been controlled on triamterene/HCTZ, 37.5/25 mg, and losartan 100 mg daily, but has increased and now also amlodipine 10 mg daily. For hyperlipidemia, takes pravastatin 10 mg daily.
- Takes ASA 81 mg daily.
- Last eye exam was on May 5, 2022, and showed no retinopathy. She has had cataract surgery also.
- Last urine microalbumin on October 14, 2022, showed microalbuminuria (high: 93)
- Denies numbness in feet or tingling, no foot ulcers
- No chest pain, palpitations, no nausea, vomiting, diarrhea, no abdominal pain, no cough or fever

Parallel semistructured interview guides were developed for patients and clinicians. Patient interviews consisted of 4 open-ended questions assessing readability, helpfulness, relevance, and anticipated future use of the SEE-Diabetes statements. Clinicians reviewed the same notes and answered 4 corresponding questions addressing clarity, completeness, clinical applicability, and suggestions for improvement. This mirrored design enabled direct comparison of perspectives across patient and clinician groups.

### Data Collection and Analysis

Participants were recruited from Family and Community Medicine clinics and the Cosmopolitan International Diabetes and Endocrinology Center in October-November 2022. Participants were asked to evaluate the Patient Education section generated via SEE-Diabetes, which was included under the assessment and plan section of the 3 clinic notes for patients (Textbox 2).

Textbox 2. **Assessment and plan section of three clinic notes. These are in-model screenshots of the Assessment and plan sections from three example clinic notes for patients with diabetes aged 65 years and older. The patient education sections were generated using Support-Engage-Empower-Diabetes, based on reviews of each patient, and then customized by an endocrinologist. Subsequently, they were reviewed by other team members. Participants were asked to review the Patient Education section and answer open-ended questions to assess the readability, helpfulness, and values of Support-Engage-Empower-Diabetes.**

**Clinic Note 1 : Assessment and Plan**

1. Uncontrolled type 2 diabetes mellitus with hyperglycemia

- Reviewed lab results with patient emphasizing the importance of optimizing HbA_1c_, with target below 8%
- Advised to check FSG regularly and record, bring records for review next visit
- Reviewed risks of hypoglycemia, prevention, and management of hypoglycemic episodes
- Reviewed foot care, call me if notice an open area on foot
- She will schedule an eye exam

**Patient Education for Monitoring**

- Monitoring is an important aspect of self-care. It helps you know if you are meeting recommended treatment goals to keep you healthy.
- My goal is to learn how to use my monitor, learn how to interpret my blood sugar levels
- I want to use this information to learn how different foods affect my blood sugar
- I commit to checking my blood sugar at the following times: 1 time a day and plan to bring in my readings to my next visit

Adapted from Association of Diabetes Care and Education Specialists (ADCES)

2. Obesity

- The patient is motivated to use weight control, which will improve metabolic health, including diabetes mellitus type 2, hypertension, and hyperlipidemia.

**Patient Education for Healthy Eating**

Discussed the meal plan today and the patient set the following goals:

- I will read the Nutrition Facts Label.
- I will add 2 servings of vegetables to my diet.
- I will cut down added sugar in my drinks from my diet to help to control my blood sugar.
- I plan to learn more about considering different healthy eating options by meeting with a diabetes specialist by the time of our next visit.

Adapted from Association of Diabetes Care and Education Specialists (ADCES)

**Clinic Note 2: Assessment and Plan**

1. Type 2 diabetes mellitus without complications

- Reviewed lab results with patient emphasizing the importance of optimizing HbA_1c_, with target HbA_1c_ below 8 %
- Advised to check FSG regularly and record, bring records for review next visit,
- Reviewed risks of hypoglycemia, prevention, and management of hypoglycemic episodes
- Reviewed foot care, call me if notice an open area on foot

**Patient Education for Taking Medications**

- Taking medications helps lower your risk for heart attack, stroke, and kidney damage by managing blood glucose, blood pressure, and cholesterol levels in your body. The longer you have diabetes, the more help you will need from medications to keep you and your heart, eyes, and kidneys healthy.
- I plan to take my medications on time by bringing in all my medications to my next appointment between now and my next visit.

**Adapted from Association of Diabetes Care and Education Specialists (ADCES)**

2. Body mass index 40+ - severely obese (finding)

- Patient has started to feel somewhat better after his COVID infection and is motivated to increase activity and control his weight to improve management of his diabetes, hyperlipidemia.

**Patient Education for Reducing Risks**

- Reducing risks means doing behaviors that minimize or prevent complications and negative outcomes of prediabetes and diabetes. Risks mean doing behaviors that minimize or prevent complications and negative outcomes of prediabetes and diabetes.
- I plan to make positive lifestyle changes, participate in diabetes self-management education.
- I will do this by scheduling an appointment by the time of our next visit.

Adapted from the Association of Diabetes Care and Education Specialists (ADCES)

- Follow-up in clinic in 3 months with labs before the appointment
- Referral placed for diabetes education again

**Clinic Note 3: Assessment and Plan**

1. Diabetes Mellitus

- Detailed discussion with the patient, reviewed HbA_1c_ of 7.2%, her target is below 8% so she is doing well. HbA_1c_ of 7.2%, her target is below 8% so she is doing well.
- However, she has gained weight and is not feeling well.
- We discussed medications that might make her mood better; however, the patient wants to focus on positive thinking first.

**Patient Education for Being Active**

- Discussed choosing favorite activities and following those fitting the patient's lifestyle. The patient identified the following goals.
- I like to walk, park farther away from the door and commit to 10 minutes daily until our next visit. This is motivating for me because I want to improve mood
- Check your blood sugar before and after exercise for safety and so you know how exercise impacts your blood sugar.

**Patient Education for Healthy Coping**

- Discussed with patient that it is important to find healthy ways to cope and not to turn to harmful habits such as smoking, overeating, drinking or alcohol. This is especially true if you have diabetes. Having a lot of stress can increase blood glucose (sugar) levels, make you feel more negative and may lead to less healthy choices.
- I plan to cope with stress by make a list of people I can turn to for support and report back at my next visit to share how that went. I will observe/record my mood daily, I will seek help if I feel challenged.

Adapted from Association of Diabetes Care and Education Specialists (ADCES)

- She will continue her current medications, focus on lifestyle and I will see her back in 3 months with labs before the appointment. She will call if she needs to make an earlier appointment.

In-depth interviews were conducted in private settings and lasted approximately 30 minutes. Sessions were audio recorded, transcribed verbatim, and de-identified. Descriptive statistics summarized participant demographics. Thematic analysis [14] was conducted using an inductive approach to identify key themes, and transcripts were coded independently by 2 researchers (PN and SD) before being reviewed by the research team.

### Ethical Considerations

This study was reviewed and approved by the University of Missouri Health Care Institutional Review Board (IRB #2078424 MU). The protocol was deemed to be no greater than minimal risk. Written informed consent was obtained from all participants, including disclosure of the study goals. Participants could opt out at any time. Nonessential identifying information has been removed for publication. Screenshots and examples included in the manuscript were deidentified so that no individual could be identified directly or indirectly. Participants were compensated with a US $50 cash card.

## Results

### Patient Characteristics and Thematic Analysis Findings From Interviews

#### Patient Characteristics

Overall, 11 patients participated, recruited from a specialty diabetes center. The average age was 72 (SD 4.9; range 66‐83) years, 6 were female (55%), and most were non-Hispanic White (10/11, 91%). Nearly half (5/11, 45.5%) had some college education. The mean duration of diabetes was 26 (SD 15; range 3‐47) years, with a mean hemoglobin A1c (HbA_1C_) of 7.6% (SD 1.2%; range 6.1%‐10.3%). Most patients were insulin users (9/11, 82%) and routinely accessed their clinic notes via patient portals (10/11, 91%), typically on their own computers (Table 1).

**Table 1.** Characteristics of patient participants (n=11).

Characteristics	Values, n (%)	
Clinic location		
Cosmopolitan International Diabetes and Endocrinology Center	11 (100)	
Age (years), mean (SD; range)	71.6 (4.9; 66-83)	
Sex		
Male	5 (45.5)	
Female	6 (54.5)	
Hispanic or Latino		
No	11 (100)	
Yes	0 (0)	
Race		
Non-Hispanic White	10 (90.9)	
Asian	1 (9.1)	
Education		
Some college credit, no degree	5 (45.5)	
Associate degree	2 (18.2)	
High school graduate, diploma, or equivalent	1 (9.1)	
Bachelor’s degree	1 (9.1)	
Trade/technical/vocational training	1 (9.1)	
Higher than a bachelor’s degree	1 (9.1)	
Diabetes duration (years), mean (SD; range)	25.6 (15; 3-47)	
HbA_1c_, mean (SD; range)	7.6 (1.2; 6.1‐10.3)	
Insulin		
No	2 (18.2)	
Yes	9 (81.8)	
Access patient portal		
No	1 (9.1)	
Yes	10 (90.9)	
How (n=10)		
Yourself	9 (90)	
With help from someone else	1 (10)	
Devices (n=10)		
Computer	8 (80)	
I appreciate the large screen (n=2)	—^a^	
It’s easy (n=2)	—	
Mobile devices	2 (20)	
My phone is always with me (n=1)	—	
Read clinic notes		
No	1 (9.1)	
Yes	10 (90.9)	

a Not applicable.

#### Readability

Most participants described the SEE-Diabetes statements as straightforward and easy to read due to plain language and clear structure. For example, a 74-year-old woman (HbA_1c_ 6.9%) highlighted that the section:

*gives you the information about any testing that you have had and the results from it*.

While an 83-year-old man remarked it was:

*well written and easily understood.*

However, some participants suggested adopting stronger motivational phrasing that better reflected a patient’s voice to encourage action, such as statements:

*to get them to take something seriously* [71-year-old man, HbA_1c_ 6.7%]

#### Helpfulness

Perceptions of helpfulness were mixed. Several participants valued the content as a practical reminder between visits*:*

*It makes it a whole lot easier… to remember what [I’m] supposed to be doing* [66-year-old woman, HbA_1c_ 10.3%]

or as a motivator to improve self-care (74-year-old woman, HbA_1c_ 6.9%).

Others, especially those with long-standing diabetes, perceived limited incremental benefit, describing the information as:

*not new* [69-year-old man, HbA_1c_ 9.2%]

or *too broad… not specific enough to make any difference* [69-year-old man, HbA_1c_ 9.2%].

One participant raised concerns about documentation practices, noting frustration with

*cut and paste… especially when the information is inaccurate* [74-year-old woman, HbA_1c_ 7%]

Overall, participants viewed helpfulness as dependent on personalization, specificity, and avoidance of redundant content.

#### Perceived Value

Patient views on added value also varied. Some appreciated the consolidation of practical information:

*They don’t have to go online and google it. The facts are here* [68-year-old woman, HbA_1c_ 6.1%]

and emphasized that SEE-Diabetes could complement physician communication, which was sometimes perceived as incomplete:

*Doctors aren’t the best at communicating all the information. I think those notes actually cover the information… better* [69-year-old man, HbA_1c_ 9.2%]

Others reported minimal added value because they were already managing well (76-year-old woman, HbA_1c_ 7.8%) or desired clearer, directive next steps:

*If there’s a diabetes education section… another section [with] recommendations… I would read that too* [71-year-old man, HbA_1c_ 6.7%]

In this context, participants referred to distinct thematic groupings within the SEE-Diabetes content, with actionable recommendations embedded under each of the 7 ADCES7-aligned headings rather than presented in a separate section. Several noted that regular updates and tailoring would be essential to maintain engagement and prevent redundancy. Additional illustrative quotes are provided in Multimedia Appendix 1.

### Clinician Characteristics and Thematic Analysis Findings From Interviews

#### Clinician Characteristics

In total, 8 clinicians participated, including 5 from specialty diabetes care clinics and family medicine (primary care) settings. The average age was 49 (SD 13.5; range 32‐65) years, and 7 were female (88%). Most were non-Hispanic White (6/8, 75%). The average experience in diabetes care was 13 (SD 12.7; range 2‐30) years. Most clinicians were familiar with ADCES7 (5/8, 63%) and DSMES guidelines (6/8, 75%) (Table 2).

**Table 2.** Characteristics of clinician participants and knowledge of diabetes self-management education and support and Association of Diabetes Care & Education Specialists 7 (n=8).

Characteristics	n (%)	
Clinic location		
Cosmopolitan International Diabetes and Endocrinology	5 (62.5)	
Keene Family Medicine	2 (25)	
Ashland Family Medicine	1 (12.5)	
Age (years), mean (SD; range)	48.6 (13.5; 32-65)	
Sex		
Male	1 (12.5)	
Female	7 (87.5)	
Hispanic or Latino		
No	8 (100)	
Yes	0 (0)	
Race		
Non-Hispanic White	6 (75)	
Asian	2 (25)	
Work experience (years), mean (SD; range)	12.7 (9.5; 2-30)	
Knowledge about DSMES^a^ and ADCES7^b^ guidelines		
Familiar with ADCES7		
No	3 (37.5)	
Yes	5 (62.5)	
Familiar DSMES		
No	2 (25)	
Yes	6 (75)	

a DSMES: diabetes self-management education and support.

b ADCES7: Association of Diabetes Care & Education Specialists 7 Self-Care Behavior.

### Clarity and Concise

Most clinicians agreed that the SEE-Diabetes statements were concise, free of jargon, and written in accessible language. A 40-year-old diabetes specialist noted that the notes “use simple language, no medical jargon, and [are] easy to read.” Similarly, a primary care physician with 2 years’ experience described the information as “short and easy to understand.” However, some clinicians highlighted areas of ambiguity. For instance, a diabetes specialist (8 y experience) observed that the phrasing around medication timing and weight control was confusing and insufficiently specific, suggesting that clearer targets, such as “work on weight loss of 5%,*”* would enhance patient comprehension.

### Sufficiency of Content

Several clinicians endorsed the adequacy of the content, describing it as “pretty thorough and self-explanatory (diabetes specialist, 8 y experience). However, others raised concerns that some sections, particularly related to medication adherence, lacked clarity and risked confusing patients. A primary care physician (2 y experience) noted difficulty interpreting the statement regarding bringing medications to the next appointment, whereas another clinician emphasized the importance of ensuring that each educational category adequately addressed patient priorities.

### Clinical Usefulness

Clinicians generally recognized the clinical utility of SEE-Diabetes in supporting patient education and reinforcing self-care. Several reported that the tool aligned with common teaching practices, such as educating patients about blood glucose monitoring, interpreting results, and linking lifestyle behaviors with outcomes (diabetes specialist, 8 y experience). Others saw potential value in emphasizing diabetes-specific goals during visits that are often crowded with competing priorities (primary care physician, 30 y experience). Nonetheless, some cautioned that time constraints may limit consistent use in busy practices. Additionally, suggestions for refinement included offering more concrete examples, such as defining portion sizes in relatable terms (diabetes specialist, 22 y experience), to maximize patient engagement and comprehension. Additional illustrative quotes are provided in Multimedia Appendix 1.

## Discussion

### Principal Findings

This study validated the content of SEE-Diabetes, an EHR-integrated patient education tool designed to support self-management among older adults with diabetes. By incorporating both expert review and direct feedback from patients and clinicians, we assessed the clarity, relevance, and clinical utility of the educational content. Our findings indicate that SEE-Diabetes has strong potential to address documentation and communication gaps in delivering DSMES and to facilitate more personalized, actionable communication during routine outpatient care. Importantly, SEE-Diabetes is not intended to replace formal DSMES, which remains an ongoing, person-centered process grounded in the assessment of individual learning needs and preferences. Participants may have received varying levels of diabetes education through prior DSMES or routine clinician-provided counseling; however, the amount and modality of such education were not assessed. Accordingly, SEE-Diabetes was evaluated as a complementary, EHR-integrated tool to reinforce routine self-management communication rather than as a measure of DSMES exposure or delivery.

Content validation was conducted using a multimethod approach that combined expert opinion, end-user perspectives, and alignment with the ADCES7 framework [15]. This strategy ensured SEE-Diabetes is grounded in scientific evidence and the practical realities of diabetes care. While content validation is sometimes overlooked in digital health tool development, it plays a critical role in ensuring safety, relevance, and usability. For instance, Patel et al [16] created a clinical decision support system for patients with serious mental illness and diabetes but relied mainly on in silico validation due to the complexity of real-world testing. Such computational methods are useful for assessing technical performance; however, they can delay clinical implementation and may overlook usability issues in practice [17]. In contrast, our study prioritized real-world applicability by engaging both patients and clinicians in the evaluation process, thereby strengthening the credibility and adaptability of SEE-Diabetes in routine care.

Readability and understandability of the educational content emerged as a central theme in the feedback from both patients and clinicians. This aligns with prior evidence that older adults, who may experience cognitive decline or limited health literacy, benefit significantly from materials presented in straightforward, jargon-free language [18]. Communicating health information in clear, familiar terms (eg, using plain language and avoiding medical jargon) significantly improves comprehension and engagement [18]. Participant feedback in our study consistently reinforced the value of plain language in promoting understanding, highlighting the ongoing need for patient-centered communication strategies across health care settings [19]. Ensuring educational content is easily digestible is especially critical for older adults, as it can empower them to more actively participate in their care.

Clinicians viewed SEE-Diabetes as a concise, efficient tool for delivering self-care guidance in time-constrained clinic visits, consistent with prior research showing that brief, targeted educational interventions can be effective in busy health care environments [20-22]. At the same time, some clinicians suggested further refining certain statements (particularly in the “Taking Medication” domain) to enhance clarity and better motivate patients. For example, one provider commented, “Bringing meds to the visit does not ensure the patient will take them regularly between visits.*”* Such feedback underscores the importance of iterative development and continuous user input to ensure that tools like SEE-Diabetes remain clinically relevant, context-sensitive, and adaptable [23]. Incorporating provider and patient suggestions in subsequent revisions will help address these nuances and improve the tool’s effectiveness.

Our analysis also identified a remaining gap in the delivery of patient-centered education during routine diabetes follow-up visits. This finding echoes prior studies indicating that although DSMES is widely implemented, it often lacks the personalization necessary to meet individual patient needs [6,7,11,24]. In our previous work, we observed that standard follow-up clinic notes frequently lacked patient-centered education for patients with diabetes [7]. SEE-Diabetes directly addresses this gap by embedding personalized educational content directly into the clinic note (which nearly 80% of our older patients reported reading via the patient portal [7]). By aligning educational messages with each patient’s unique context and self-management goals, this approach supports the broader movement toward patient-centered care. Such individualized interventions are expected to enhance patient engagement and treatment adherence and ultimately improve outcomes in diabetes management.

### Strengths and Limitations

A key strength of this study lies in its user-centered validation approach, which engaged both patients and clinicians across primary care and specialty care settings. By involving real-world end users in the design and evaluation process, we ensured that SEE-Diabetes content is not only evidence-based but also practical, readable, and clinically relevant. The use of tailored clinical scenarios, combined with in-depth qualitative interviews, provided rich insights into the clarity, usefulness, and perceived value. This multistakeholder engagement enhances the credibility of our findings and supports the tool’s adaptability across diverse workflows, thereby strengthening its potential for real-world implementation. Notably, our approach aligns with UCD principles that emphasize iterative development and continuous involvement of target users [9]. By continuously incorporating feedback from both providers and patients, we aimed to develop an educational tool that meets users’ needs in everyday practice.

Limitations of this study include a small sample size and a lack of racial and geographic diversity in our participants. Because the majority of participants were non-Hispanic White and recruitment was limited to a single academic health center, the generalizability of our findings may be constrained. This homogeneity is consistent with the demographic profile of the Midwestern United States, where approximately 73% of the population identifies as non-Hispanic White, which likely influenced the composition of our sample [25]. Future work should evaluate SEE-Diabetes in larger and more diverse populations and test its implementation across various clinical settings and regions. Despite these limitations, our study supports the feasibility and potential value of integrating personalized education into routine care through tools like SEE-Diabetes. The structured, user-informed content provided by SEE-Diabetes may help improve patient-provider communication, support patient self-management, and ultimately contribute to more patient-centered chronic disease care.

### Future Directions

Beyond the current implementation, SEE-Diabetes has potential for broader scalability across diverse care settings. While this study focused on EHR-based delivery, future work could explore parallel formats such as printable summaries or patient-facing handouts to support clinics without advanced EHR functionality, including rural and resource-limited programs. Additionally, situating SEE-Diabetes within national DSMES Standards and the 4 critical times for DSMES delivery may help align its use with formal education pathways while reinforcing self-management communication during routine care.

### Conclusions

This study validated SEE-Diabetes, a patient-centered tool that embeds tailored diabetes self-management support into EHR notes for older adults. Both patients and clinicians confirmed that the content is clear, relevant, and feasible for integration into primary and specialty care. Embedding plain-language education within routine documentation may strengthen communication, reinforce self-care, and support chronic disease management in aging populations. Future work should evaluate implementation across diverse settings and its impact on clinical outcomes, engagement, and scalability.

## Supplementary material

10.2196/83448 Multimedia Appendix 1 Representative participant quotations illustrating perceptions of clarity, relevance, usability, and clinical usefulness of SEE-Diabetes content, organized by stakeholder group (patients and clinicians) and ADCES7-aligned domains.

## Abbreviations

ADCES7: Association of Diabetes Care & Education Specialists 7 Self-Care Behaviors
DSMES: diabetes self-management education and support
EHR: electronic health record
SEE-Diabetes: Support-Engage-Empower-Diabetes
UCD: user-centered design
