# Supplementary material for: Content Validation of an Electronic Health Record–Based Diabetes Self-Management Support Tool for Older Adults With Type 2 Diabetes: Qualitative Study
Source: JMIR Diabetes. 2026 Feb 6;11:e83448. doi: 10.2196/83448 (PMC12880848; doi:10.2196/83448)
Supplement: Multimedia Appendix 1 [file diabetes-v11-e83448-s001.docx]

**Examples of patients’ statements on the readability, helpfulness, and perceived value of the Patient Education section.**

| **Patients’ perspectives** | | | |
| --- | --- | --- | --- |
|  | **Clinic Note 1** | **Clinic Note 2** | **Clinic Note 3** |
| **Readability** | “Yes, it’s easy to understand and easy to read. It usually gives pretty good details as to what your doctor’s report is. I like the idea that it gives you out education to let you know that she is actually looking at your status and your history. It just basically gives you the information about any testing that you have had and the results from it.” *- 74-year-old Female, A1C 6.9%, diabetes duration 45 years* | “Yes, I understand this. One of the things that maybe need to be in this education section is a statement that’s almost by a patient to get them to take something seriously.” *- 71-year-old Male, A1C 6.7%, diabetes duration 47 years* | “It’s well written and easily understood.” *- 83-year-old Male, A1C 8.2%, diabetes duration 30 years* |
| **Helpfulness** | “Very, very helpful. Because it’s easy to sit in the doctor’s office and hear what he has to say, but remembering that later it’s sometimes hard, so if you have this, you can look back and see. This is what we’re supposed to do. And so, it makes it a whole lot easier for a person to remember what they’re supposed to be doing.” *- 66-year-old Female, A1C 10.3%, diabetes duration 3 years* | “It is helpful. One of my biggest issues with the way charting is done is cut and paste, but you know, I really hate that, especially when the information is inaccurate and you make a point about it hasn’t been changed throughout the years, you know, that’s one, and that may not relate to this, but that’s one particular problem that I have with the documentation.” *- 74-year-old Female, A1C 7.0%* | “It is helpful because it gives her the fact that she was able to discuss this with her doctor. To encourage and motivate you to do better.” *- 74-year-old Female, A1C 6.9%, diabetes duration 45 years* |
|  |  | “A little bit. I mean, I have had diabetes for a long time, so this is not new or anything.” *- 69-year-old Male, A1C 9.2%, diabetes duration 5 years* | “Not much. I’m not sure it tells anything. I think it’s too broad. It is not specific enough to make any difference.” *- 69-year-old Male, A1C 9.2%, diabetes duration 5 years* |
| **Perceived Value** | “I think it is going to increase the education. They don’t have to go online and google it. The facts are here.” *- 68-year-old Female, A1C 6.1%, diabetes duration 20 years* | | |
|  | “At my point in life, I don’t, you know, I’m getting along so well. I don’t think I need that so much. It would be interesting.” *- 76-year-old Female, A1C 7.8%, diabetes duration 38 years* | | |
|  | “I think it does if the doctors actually do this. Doctors aren’t the best at communicating all the information. I think those notes actually cover the information that needs to be done better.” *- 69-year-old Male, A1C 9.2%, diabetes duration 5 years* | | |
|  | “I think if there’s a diabetes education section, I would see if there’s anything I’m missing. So, the answer is I would, like you said, another section. And if it’s recommendations, I’m not sure recommendations are the right work considerations. Whatever the word is, I would read that too. I mean, I would read it.” *- 71-year-old Male, A1C 6.7%, diabetes duration 47 years* | | |

**Examples of clinicians’ statements on the clarity and conciseness, sufficiency of content, and clinical usefulness of SEE-Diabetes.**

| **Clinicians’ perspectives** | | | |
| --- | --- | --- | --- |
|  | **Clinic Note 1** | **Clinic Note 2** | **Clinic Note 3** |
| **Clarity and Conciseness** | “Yes, this note does address self-care information. This one is addressing monitoring and why it is important to what their goal is and what they’re committing to doing so that does meet that goal, number one, to communicate self-care information.” *- 64-year-old Female, diabetes specialist, work experience 8 years* | “Yes, I think, and again, in terms of reasoning, it’s not too lengthy. It uses simple language, no medical jargon, and keeping it to the point, easy to read. So, I think it serves the purpose.” *- 40-year-old Male, diabetes specialist, work experience 10.5 years* | “Yes, the information is short and easy to understand.” *- 32-year-old Female, primary care, work experience 2 years* |
|  |  | “I think this was confusing. I plan to take my medications on time by bringing in all my medications to my next appointment, and then it says between now and my next visit. And I know you are using templates, so I think it’s just not specific. It’s great that the patient understands they need to increase activity and control their weight, but do they really understand what that means to control their weight? It might be even better to say work on weight loss of 5%. Then that’s defined: what am I losing.” *- 64-year-old Female, diabetes specialist, work experience 8 years* | “Yes, addresses top priority concerns of improving activity level and encouraging social life to decrease depression. Educate about the severity of smoking.” *- 37-year-old Female, diabetes specialist, work experience 11 years* |
| **Sufficiency of Content** | “Yes, I think it’s pretty thorough and self-explanatory, and it’s measurable. So, I think that’s sufficient on that one goal of monitoring.” *- 64-year-old Female, diabetes specialist, work experience 8 years* | “No, the “Taking Medication” section is a little confusing when it states, “I plan to take my medications on time by bringing in all my medications to my next appointment between now and my next visit.” I am a little confused about this plan.” *- 32-year-old Female, primary care, work experience two years* | “All important topics and patients are addressed in 2 education categories.” *- 40-year-old Male, diabetes specialist, work experience 10.5 years* |
| **Clinical Usefulness** | “Yes, I would use it because often I do talk to patients about why it’s important to monitor. A lot of patients don’t monitor, so I’m teaching them how to use their monitor and then what their targets are, and this says learn how to interpret the levels, and so that is good that you’ve put that in there as well. And then you have to mention that it’s not just actual monitoring that they have to reflect on how the foods affect their blood sugars.” *- 64-year-old Female, diabetes specialist, work experience 8 years* | | |
|  | “It’s a matter of time. It would add a step, and you know, many times are when we take care of the diabetes. We’re also checking to let you know that you have hypertension and this and that. So, few of our visits are diabetes, we’re just going to talk about your diabetes today, and that’s, you know, that alone would probably be an important thing to make that an emphasis that this is all we’re going to talk about today is your diabetes.” *- 65-year-old Female, primary care, work experience 30 years* | | |
|  | “I am thinking kind of what like when you say serving size just more define more serve but serving size is. The size of your hand or something like that. Yeah, serving size, maybe. I don’t know it looks good to me.” *- 59-year-old Female, diabetes specialist, work experience 22 years* | | |
